# Reply to “Improving Microbiology Research: the Problems Are Less Statistical and More Biological”

**DOI:** 10.1128/mBio.01680-16

**Published:** 2016-10-18

**Authors:** Arturo Casadevall, Lee M. Ellis, Erika W. Davies, Margaret McFall-Ngai, Ferric C. Fang

**Affiliations:** a Department of Molecular Microbiology and Immunology, Johns Hopkins Bloomberg School of Public Health, Baltimore, Maryland, USA; b Division of Surgery, Department of Surgical Oncology, University of Texas MD Anderson Cancer Center, Houston, Texas, USA; c American Society for Microbiology, Washington, DC, USA; d Pacific Biosciences Research Center, University of Hawaii at Manoa, Honolulu, Hawaii, USA; e University of Washington School of Medicine, Seattle, Washington, USA

## REPLY

We thank Dr. Lawler for his letter (1), which argues that a root cause of the problems in science lies in our educational system. We are in general agreement with his comments and concerns. Our report (2) was the result of a colloquium organized by the American Academy of Microbiology that focused on specific questions relating to research reproducibility, not all possible problems with bioscience research and publication. Nevertheless, our report does recommend an increased emphasis on improving didactic training of scientists in basic methods of research. Although the specific recommendations regarding education differ somewhat from those of Dr. Lawler, there is considerable harmony in the spirit of the recommendations made by colloquium participants and his correspondence.
